# What is the risk of a deadly adenovirus pandemic?

**DOI:** 10.1371/journal.ppat.1009814

**Published:** 2021-09-02

**Authors:** Eric J. Kremer

**Affiliations:** Institut de Génétique Moléculaire de Montpellier, Université de Montpellier, CNRS, Montpellier, France; Boston Children’s Hospital, UNITED STATES

## Abstract

Many of us had refresher courses in virology, immunology, and epidemiology in 2020, and we were reminded of the fact that *Homo sapiens*, the wiliest predator on the planet, has been hunting everything that moves for millennia. These repeated interspecies contacts inherently lead to recurrent zoonosis (nonhuman to human) and anthroponosis (human to nonhuman). Given the accelerating changes in our ecosystems since the neolithic revolution, it was not surprising to see a virus that spreads via aerosolization and liquid droplets cause a pandemic in a few months. The Severe Acute Respiratory Syndrome Coronavirus 2 (SARS-CoV-2) pandemic begs the question—which viruses could cause a global threat? In this Opinion, the characteristics that make adenoviruses a risk, which include efficient intra- and interspecies transmission, thermostable particles, persistent/latent infections in diverse hosts, and the ability to readily recombine and escape herd immunity, are discussed.

## Introduction

What combination of viral and host characteristics will favor the next pandemic? A prioritized checklist might include (i) efficient transmission by aerosolization; (ii) asymptomatic spreaders and latent infections that foster a high reproductive number (R factor); (iii) the aptitude of the pathogen to generate “variants” that circumvent herd immunity; (iv) no available drugs able to prevent virus propagation or disease; (v) a thermostable particle resistant to disinfectants and that can remain infectious for days while on tissue, metal, or plastic; (vi) multiple and highly conserved portals (receptors) to infect cells and tissues; (vii) propensity of the virus to be endemic in settings that then foster rapid regional and intercontinental spread; and (viii) susceptibility to cyclic zoonotic and anthroponotic exchanges that favor large and diverse reservoirs. Adenovirus fulfills these requisites. Of note, this list is not exhaustive, nor are all the factors essential.

### A fertile environment for propagation

In the 1950s, Rowe and colleagues were culturing cells from human adenoids that underwent spontaneous degeneration [1]. From these cultures, they isolated an adenovirus. Adenoviridae is a family of nonenveloped icosahedral particles of approximately 90 nm in diameter and contains a double-stranded linear DNA genome of 26,000 to 48,000 bp. Like many other viruses, adenoviruses encode multitasking structural and regulatory proteins. These viral proteins interact with the host proteome and genome to allow receptor-mediated uptake, endosomal escape, intracellular trafficking, genome replication and transcription, preferential translation of viral mRNA, genome encapsidation, and release of metastable virus particles. Adenoviridae is found on every continent and now includes more than 300 officially recognized types isolated from mammals, fish, birds, and reptiles [2]. Adenoviruses that infect humans (human adenoviruses [HAdVs]) belong to the genus *Mastadenoviridae* and are grouped into 7 species (A to G), which include approximately 100 types that are categorized by serology and/or sequence phylogeny (http://hadvwg.gmu.edu).

Anecdotal reports suggest that most of the approximately 160 *Mastadenoviridae* can be propagated (some up to 50,000-fold amplification/cycle/cell) in cell culture, readily purified, concentrated, and stored long term. These characteristics led to their use as molecular and cellular biology tools throughout the 1970s and 1980s to complement biochemical and structural analyses. Using adenoviruses as a model system, pioneering studies in DNA replication and RNA splicing helped establish the bases of modern molecular and cellular biology [3,4]. Yet, there is still much to be discovered. For example, at the molecular level greater than 800, alternatively spliced mRNAs from a HAdV have been recently annotated [5].

With respect to our immune response to HAdVs, epidemiological data generated during the last 6 decades suggest that, similar to some α- and β-coronaviruses, greater than 90% of us have been infected by a handful of HAdV types by the time we are a few years old [6]. Most HAdV infections cause type-specific, asymptomatic, self-limiting disease in the respiratory tract, conjunctiva, and/or gastrointestinal tract. The incubation period is typically 5 to 8 days, and shedding can continue for several weeks [7]. Data from healthy and immunosuppressed individuals are consistent with the idea that some HAdVs induce latent/persistent infections that last for decades [8]. Persistent infections are also coherent with clinical data showing that in patients undergoing pharmaceutical or disease-induced immune suppression, adenoviremia can be traced to the seroprevalence of the same type before immune suppression [8].

By most measures, the humoral and cellular immune responses to HAdV infections are multifaceted and robust [9–11]. Importantly, we are only beginning to understand how extracellular proteins (e.g., antibodies, coagulation factors, complement, and host defense proteins) influence the innate response of antigen-presenting cells [12,13]. HAdV type–specific antibodies are typically generated following vaccination or infection. Because most HAdV infections of civilians are self-limiting, immune correlates for protection have not received much attention. However, it is worth noting that in military recruits, HAdV-E4 neutralizing antibody (NAb) titers >1:32 are typically protective (n.b., some recruits with HAdV-E4 NAbs are nonetheless hospitalized with acute respiratory disease (ARD) [14,15]. Interestingly, cross-reactive antibodies can, in some cases, provide protection against HAdV disease [16]. While protection provided by cross-reactive antibodies has not been thoroughly examined, vaccination with HAdV-E4 and -B7 does provide protection against HAdV-B14. In addition, Russell and colleagues found that individuals who harbor naturally occurring (i.e., not by vaccination) HAdV-B7 NAbs are less likely to develop HAdV-B14–associated disease during an outbreak in a military training facility [7]. These observations complicate the dogma surrounding 50 years of epidemiology studies based on the idea that we develop type-specific NAbs following HAdV infections. It is likely that protective, heterotypic B cell responses may be efficient against HAdV from very similar types (i.e., humoral responses against HAdV-B7 protect against HAdV-B14 but not vice versa or against HAdV-B35).

In addition to a humoral response to HAdVs, long-lived memory T helper type 1 (Th1) cellular response is found in all cohorts. Notably, *Mastadenoviridae* contain highly conserved regions in the internal base of hexon that are targets of cross-reactive memory T cells, which are likely refined at each encounter with a different HAdV type [17,18]. The ability to amplify anti-HAdV memory T cells ex vivo, and then infuse these cells to patients to treat adenovirus disease, is a life-saving therapy for some individual undergoing solid organ or bone marrow transplants [19].

Yet, several times a year, HAdV outbreaks cause severe disease or death in a population of healthy civilians in resident care facilities, prisons, or military training camps [20]. For example, HAdV-B3 and HAdV-B7 cause repeated outbreaks of ARD in China. After decades of being prevalent in Eurasia, HAdV-B14 (isolated in a military training camp in the Netherlands in the 1960s) appeared in the United States near 2005, killing 18% of the 38 cases in Oregon. By 2015, HAdV-B14 had become endemic in US military training facilities and caused sporadic epidemics in civilian populations. Typically, outbreaks in healthy populations disappear within several weeks. While these events are sporadic, severe HAdV disease is a constant and lethal threat in immunocompromised individuals undergoing hematopoietic stem cell or solid organ transplants [8]. Factors such as lack of preexisting immunity, physical and mental stress, and overcrowding increase the risk of infection with respiratory pathogens. Environments where these factors exist include civilian and military training facilities, staff and patients at long-term psychiatric care facilities, children in day care, and elderly individuals and staff at chronic care facilities centers. These scenarios also bring to the front a dichotomy that is difficult to reconcile: In individuals with intact B-cell functions, but compromised T-cell responses, HAdV infection can lead to fulminant and fatal disease [8]. In addition, treating patients with intravenous immunoglobulin (IVIg) (purified immunoglobulin Gs (IgGs) from 5,000 to 10,000 individuals), which contains NAbs against many HAdV types, has mixed success against advanced HAdV disease.

### Receptor options and cytoplasmic trafficking

The thousands of replication-defective human and nonhuman adenovirus vectors generated over the last 40 years have been invaluable tools to understand adenovirus uptake in mammals, fish, amphibians, and birds. Using naturally occurring capsids, or taking advantage of their LEGO-like structural adaptability to swap proteins and motifs from other types, our understanding of adenovirus receptor engagement is rich in detail [21]. The options for an adenovirus finding a cell surface entity that can be used as an initial port of entry include highly conserved cell adhesion molecules, complement receptors, lectin-binding proteins, ubiquitous heparan sulfate proteoglycans, and sialic acid–tagged proteins [22]. Other opportunities for cell engagement include interactions with soluble extracellular proteins (e.g., opsonizing antibodies, vitamin K–dependent coagulation factors, antimicrobial peptides, and complement components system) that act as bridges to other ubiquitous cell surface entities (e.g., Toll-like receptors) [23], which diversify the options for engagement and internalization. I posit that either via direct receptor engagement or via an appropriate soluble extracellular protein, most adenoviruses can be internalized to some extent by most nucleated mammalian cells.

Are there species-specific restriction factors that could prevent adenovirus receptor-mediated internalization, escape from the endosome, trafficking through the cytoplasm, docking at the nuclear, and delivery of their genome into the nucleus? Adenoviruses use highly conserved cellular transport mechanisms along microtubule tracks to reach the nuclear pore, where they bind and deliver their genome to the nucleus [21]. No (efficient) host restriction factor precludes adenovirus uptake by human, monkey, ape, dog, sheep, cow, rodent, or bird cells in vitro or in vivo.

### Recombination and transcription

One characteristic favoring virus longevity is the constant production of mutants that can endow selective advantages. A source of mutants is, in part, the lack of fidelity of the viral polymerase. Viruses with an RNA genome, such as influenza and coronaviruses, tend to accumulate mutations relatively quickly, and these mutations provide opportunities to escape preexisting immunity. Yet, at 1.3 × 10^−7^ mutations/bp, the adenovirus polymerase (assuming all are similar to HAdV-C2) is relatively high fidelity. Furthermore, essentially identical genomes of some adenovirus types can be found decades apart [24]. While a high-fidelity polymerase limits genetic drift, recombination provides a pathway to generate new adenoviruses with altered tissue tropism and the ability to escape host defenses [25,26]. It is worth repeating that at the end of the roughly 36-hour replication cycles, there are greater than 50,000 copies of the adenovirus genome/cell. If 2 adenovirus types are found within the same cell, the probabilities of recombination events, even within short stretches of homology, are significant.

As mentioned above, persistence and latency also provide an ideal milieu for the encounter of multiple HAdV types in a host. Due to increased efficacy of broad-range PCR, the number of “novel” human and nonhuman adenovirus types is rapidly expanding [2]. A study in which whole-genome sequence analyses were used to discriminate, type, and characterize HAdVs supports the idea that homologous recombination is responsible for the genesis of numerous types with distinct virulence. The fastest expanding clades are those from those of *Mastadenoviridae* species B and D [25,26]. An example of increased virulence is HAdV-B55, in which HAdV-B14 acquired part of the hexon open reading frame (hexon is the major structure outer capsid component and targeted by NAbs) from HAdV-B11. HAdV-B55 escapes preexisting immunity and has caused severe respiratory disease in individuals in hospitals, schools, and military training facilities [27]. Of note, increased virulence of some “new” types is also due to changes in tissue tropism, which may be linked in some cases to the homing of memory T cells [28].

### Host infidelity

**Fig 1:**
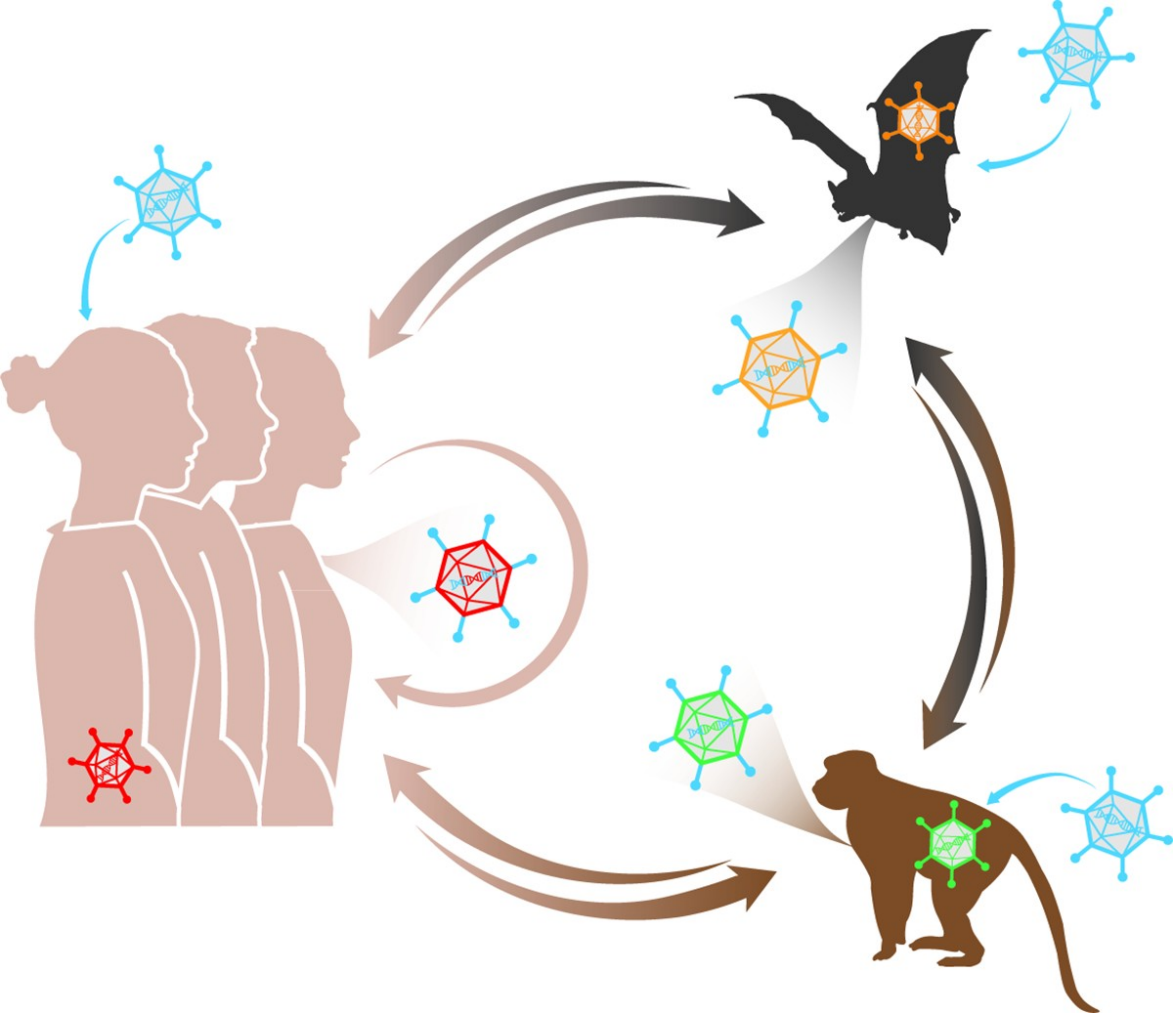
Many mammals have latent adenoviruses (red capsid in humans, green in monkeys, orange in bats) that are controlled by anti-adenovirus memory T cells in healthy hosts. These viruses may have replicated and induced asymptomatic or mild disease in the respiratory or gastrointestinal tract. Notably, in primates, adenovirus types that generate respiratory tract infections are often shed via the gastrointestinal tract. When a host encounters another type (blue capsids) that propagates in the same tissue, the opportunity for recombination increases. If a recombination event gives a selective advantage (e.g., escape of immune surveillance, modified tissue tropism, and alternative receptors) to the new type (multicolored capsids), it can be passed to other individuals to reinitiate the same scenario. Mutations that increase the host range (e.g., in the DBP) also occur randomly and expand the reservoirs and increase the propensity for zoonotic and anthroponotic exchanges—and the creation of more virulent types. DBP, DNA-binding protein.

The volume of interactions between humans and animals is increasing in many areas. Mass production of livestock in poor hygienic conditions reduced antigen exposure in humans due to urbanization, megacities, and affordable international travel, abet zoonosis, and anthroponosis (**Fig 1**). Next-generation sequencing has uncovered an extraordinary diversity of adenovirus types and has led the field to reexamine origin, diversity, host range, and epidemiological data. It appears probable that human behavior over the last 200 years has had a greater influence on adenovirus evolution than the previous 10,000 years, but this is difficult to prove. An analysis by Bayesian molecular clock dating could, potentially, address the issue of adenovirus evolution [29]. In addition, considering what has been discovered regarding the interspecies transmission of canine, simian, and HAdVs, it is not inconceivable that what has previously been interpreted as “species specificity” might actually reflect the lack of contact rather than an inability to infect related species [30].

During the last 60 years, conclusions based on epidemiological analyses led to the dogma that most members of *Mastadenoviridae* have a host range limited to a single or closely related species. Ockham’s razor favors the notion that regulatory proteins encoded by the early region 1 (E1) (which encodes transactivating factors and proteins that inhibit apoptosis or modify the cell cycle) and E4 (which encodes proteins involved in transcription, mRNA processing and transport, cell cycle, cell signaling, and DNA repair) limit host switching [31]. This is because viral and host proteins interact in defined orientations, and deviations in protein structure, charge, and subcellular location will preclude efficient virus propagation. This rationale is embodied by the E1a protein from HAdV-C2 [32], which may interact with 200 human proteins.

Our improved understanding of *Mastadenoviridae* diversity and host origin is, paradoxically, due in part to the search for, and sequencing of, nonhuman primate adenoviruses as potential vaccine vectors [33] (see Projection). At the moment, the only *Mastadenoviridae* species E member found in humans (HAdV-E4) falls within a gorilla/chimpanzee clade [2]. Phylogenetic analyses also place the 16 original HAdV***-***B types as pathogens originating in great apes [34]. Inexplicably, HAdV-E4, HAdV-B7, and HAdV-B14 are endemic in many military training centers in the USA, China, Turkey, Canada, Finland, and the Netherlands and cause unusually severe disease. The factors that allowed HAdV-E4 and HAdV-B7 to impact military training centers, which are home to typically healthy 18- to 27-year olds for approximately 12 weeks, are poorly understood and not unique to adenoviruses. To prevent the loss of strategic military readiness, recruits in several countries were vaccinated with live oral HAdV-E4 and HAdV-B7, which significantly reduced disease at the facilities for 25 years. When the vaccine stocks were exhausted in 1999, HAdV-E4 and HAdV-B7 disease frequency erupted again in a couple of years. Was the “remontada” due to shedding by asymptomatic training staff? Or was it due to reintroduction by new recruits? Of note, greater than 98% of recruits were seronegative at enrollment [7]. Importantly, the study was unable to find active HAdV-E4 infections in the onsite staff or recruits on day 1. At the centers tested, all trainees became HAdV-E4 seropositive within 6 weeks.

In addition to HAdV-E4 and HAdV-B7, there are widespread anecdotal and specific [35,36] examples of adenovirus exchanges between simians and humans. A Titi monkey adenovirus caused a virulent outbreak in the monkey colony and also infected a member of the research staff [36]. The researcher then spread the simian virus to family members. Medkour and colleagues recently concluded that many additional *Mastadenoviridae* species C types are endogenous in gorillas and humans (there are currently 5 types found routinely in humans) [37]. Taking into account species infidelity and recombination, Seto and colleagues proposed that HAdV-B76 was generated from recombination events of a virus that infected humans, chimpanzees, and bonobos [25]. At the apex of host range infidelity may be the 2 canine adenovirus types (CAdV-1 and CAdV-2). CAdV-1 and CAdV-2 infect dogs and wolves of course, but antibodies can also be found in jackals, foxes, coyotes, racoons, skunks, bears, pandas, sea lions, walruses, fur seals, and dolphins [38,39]. More ominous is that phylogenetically CAdVs fall in the middle of a bat adenovirus clade [40].

If there is a candidate for host switching, the 72-kDa DNA-binding protein (DBP) should get special attention. DBP is required for the initiation and elongation of the adenovirus genome and was identified during the initial characterization of DNA replication in the 1970s [41]. Its role in replication is consistent with the approximately 10 million copies produced/infected cell. While DBP was methodically examined at the functional levels over 40 years ago, the structure of the nucleic acid–binding carboxyl terminus could only be solved when the intrinsically disordered 20-kDa N terminus was removed [42]. The DBP enigma was born when HAdV-C2 and HAdV-C5, which efficiently grew only in human cells, were serially passed on monkey cells. Eventually, a handful of “host range” mutants broke through [43]. The remarkable result was that each mutant had a histidine to threonine switch in the N terminus of DBP (at amino acid [aa] 129) [44]. It is also noteworthy that the N terminus can be phosphorylated at 15 sites, which create a potential combination of greater than 32,000 forms)—and likely plays a critical role in the formation of membraneless virus replication centers (VRCs) via liquid–liquid phase separation [45]. The formation of supramolecular structures is caused by proteins containing intrinsically disordered regions made up of short linear interaction motifs, alternating charge blocks, or degenerate repeats [46]. It is therefore reasonable to propose that DBP plays a fundamental role in the forces that drive VRC formation—and ipso facto genome replication. How do mutations in DBP favor host switching though? While there has been some success with the isolation of VRCs, their composition is still unresolved. Opposing mechanisms could lead to the same downstream effect: The addition of a phosphorylatable threonine at aa 129 allows the recruitment of factor(s) in monkey cells that favor(s) replication, transcription, or RNA transport. Conversely, the mutation prevented the recruitment of adenovirus restriction factor(s) to the VRC that favored replication, transcription, or RNA transport. The list of possibilities is vast.

### Projection

The recurrence of pandemics has accelerated in the last 150 years: 1 in the first half of 20th century (1918 influenza pandemic), 3 in the second half (Asian flu, Hong Kong flu, and AIDS), and now 4 in the first 20 years of 21st century (SARS, H1N1 “porcine” flu, Zika, and Severe Acute Respiratory Syndrome Coronavirus 2 (SARS-CoV-2)). At the beginning of the current pandemic, many virologists were not surprised that another coronavirus appeared—only that it was so virulent. It will not escape the attention of seasoned virologists that all of the above are RNA viruses. While DNA viruses are typically considered an unlikely source as pandemic-provoking pathogens, primarily because of the fidelity of their polymerases, adenovirus variants can be generated via other pathways. Although some would be surprised to see an adenovirus pandemic, there are fertile ecosystems that cannot be ignored. As delineated in the introduction, *Mastadenoviridae* constitute an exception among DNA viruses. Still, skepticism is not without merit. If adenoviruses are a threat, why hasn’t it happened before? Have humans escaped an “adenovirus pandemic”? Based on seroepidemiological data of some HAdV types (like the species C members), my conclusion is “no.” Have we escaped a “deadly” pandemic”? Yes, for now. Is there a missing trigger? Do we need more stress? A breakdown of public health systems? A chance encounter with a bat or a nonhuman primate? Paradoxically, as the adenovirus-based (HAdV-C5, HAdV-D26, and chimp adenovirus ChAdOx1) SARS-CoV-2 vaccines are being deployed [33], we will need to take into account their long-term impact on zoonosis and anthroponosis and our susceptibility to nonhuman primate adenoviruses. Will these vaccines prevent, have no impact, or foster the emergence of an adenovirus pandemic? Some HAdV-C5, HAdV-D26, and ChAdOx1 vaccine genomes will be taken up by circulating T cells—the same cells where one finds latent adenoviruses. Will this be the one-in-a-million chance trigger? On the other hand, what better vaccine platform exists than a replication-defective adenovirus vector to combat an adenovirus pandemic?
